# Polypropylene Random Copolymer Based Composite Used for Fused Filament Fabrication: Printability and Properties

**DOI:** 10.3390/polym14061106

**Published:** 2022-03-10

**Authors:** Zhiyao Zhang, Xueqin Gao

**Affiliations:** 1 School of Aeronautic Science and Engineering, Beihang University, Beijing 100191, China; zhangzhiyao2001@buaa.edu.cn; 2 College of Polymer Science and Engineering, Sichuan University, Chengdu 610065, China

**Keywords:** fused filament fabrication, polypropylene random copolymer, printability, toughness, composites

## Abstract

Fused filament fabrication (FFF) is one of the most commonly used additive manufacturing technologies. However, the applied material for commercial FFF is limited. Presently, though being one of the most used polymer materials, polypropylene (PP) is rarely used in FFF because of its serious warpage and shrinkage problems. This study investigated the impact of addition of short glass fibers (GF) and ethylene propylene diene monomer (EPDM) on the printability of polypropylene random copolymer (PPR) based FFF and mechanical properties of the printed samples, as well as other properties including rheology, thermal behaviors, and morphology. The results show that the modified PPR has excellent printability, as the printed samples are of good geometrical accuracy. The addition of GF can significantly improve the strength and modulus of the composite, but it also leads to serious decrease in toughness. EPDM addition can effectively improve the toughness of the composite, showing a complementary effect with GF. This work has important meaning in expanding the FFF applicable material and in broadening the application of PP.

## 1. Introduction

Additive manufacturing (AM), also known as 3D-printing technology or rapid prototyping technology, has been developing rapidly since it was first proposed in 1986 [1,2] because of its unique advantages. AM allows for more customized characteristics and can significantly simplify the process of prototyping, making the production more efficient [3]. In addition, it has little raw material waste in production [4] and, therefore, it is eco-friendly. As a result, AM is nowadays widely used in different areas [5,6,7], such as aeronautics, medical equipment, architecture, car industry, etc., and its application is still expanding.

After decades of advances, AM has developed into different types [8,9,10,11], including selective laser sintering (SLS), stereolithography (SLA), Laminated Object Manufacturing (LOM), and fused filament fabrication (FFF), etc. Among them, FFF is one of the most widely used methods for fabricating thermoplastic parts with the advantages of low cost, minimal wastage of raw material, and ease of material change [3,12,13,14]. The FFF process can be described as follows [15,16]. A thermoplastic filament with uniform diameter, which is used as the printing material, is fed into a heated extrusion head. It then melts and is pushed out of a nozzle. The extruded melt deposits on the build platform. At the same time, the head moves horizontally to change the position of the deposition, and the build platform moves downwards once a layer has been finished so that the next consecutive layer can be deposited. The printed object is modeled with the superimposition of layers. Presently, materials such as acrylonitrile butadiene styrene (ABS) and polylactic acid (PLA) are commonly used for commercial FFF production [17,18]. However, FFF still has an unignorable problem: some polymers, such as polyolefin, are barely used for commercial FFF despite their ideal properties and broad applications.

Polypropylene (PP) is a versatile material that has numerous applications because of its good mechanical and biological properties, thermal stability, chemical inertness, and cheapness [19,20]. These properties make PP a good candidate for FFF. However, because of the semi-crystalline nature of PP, the fabricated sample tends to shrink and warp seriously during the printing process, causing acute inaccuracy or even failure in printing [21,22].

There are several types of PP. Among them, isotactic polypropylene (iPP) is the one most studied because of its comprehensive properties and abundant crystal modifications [23,24]. In recent years, research focusing on improving the printability of iPP has been conducted. Carneiro et al. investigated the effect of printing orientation, infill degree, layer thickness, and addition of glass fiber on the FFF printed parts and presented a comparison to the injection molding method [25]. In addition, 30% and 40% increased modulus and strength, respectively, were observed with the 30% glass fiber filler. However, they did not report the influence of GF on the printability of iPP. Gholamhossein et al. also studied the influence of glass fiber fillers on the properties of iPP [26]. They found that GF could bring better adhesion between platform and parts, as well as improved strength and reduced flexibility. The enhanced adhesion could to some extent improve the printability of iPP. Spoerk et al. investigated the system of expanded-perlite filled iPP [27]. Shrinkage analysis was conducted and results showed that a 25% reduction of deformation was achieved with the optimized filler content and filler diameter. Enhanced toughness and reduced stiffness were also observed with the expanded-perlite filler. Leng et al. [15] and Bachhar et al. [28] reduced the warpage deformation of printed parts by enhancing the adhesion between the parts and the printing platform.

Besides iPP, Polypropylene random copolymer (PPR) is also a widely used type of PP [29,30]. It is prepared by copolymerization of propylene and ethylene comonomer. The crystallization of propylene is disrupted by the randomly embedded ethylene units [31]. With the lower crystallinity, PPR has better toughness and a lower melting point compared with iPP. In addition, a reduced deformation can be expected because of the lower crystallinity. Hence, we believe PPR is more suitable for FFF than iPP. However, to the best of our knowledge, there is very little research about this topic.

In the current work, we try to improve the geometrical accuracy of a printed sample by adding short glass fibers (GF) and ethylene propylene diene monomer (EPDM) into the PPR matrix to make it printable for FFF, and, at the same time, enhance the toughness of material. The printability and properties of the composite were characterized by mechanical tests, thermogravimetric analysis (TGA), differential scanning calorimetry (DSC), high pressure capillary rheometer, scanning electron microscope (SEM), and optical warpage analysis. The resulting sample displayed good geometrical accuracy, as well as significantly improved toughness. The study can broaden the applicable materials for FFF.

## 2. Materials and Methods

### 2.1. Materials

PPR (R200P) was provided by Hyosung Corporation, Seoul, South Korea. Its melt flow rate (MFR) is 0.25 g/10 min (230 °C/2.16 Kg), and weight average molecular weight (M_w_) is 7.2 × 10^5^ g/mol. The xylene monomer content is 3.8%. Short glass fiber (ESC13-4.5-508A) was provided by Jushi Group Co., Tongxiang, China. Its diameter and length are about 13 μm and 4.5 mm, respectively. EPDM (2032 pm) was provided by Mitsui Chemical lnc., Tokyo, Japan. All of the raw materials were used as received.

### 2.2. Sample Preparation

In this experiment, the ratio of PPR and GF was fixed at 6:4. They were then melt blended with different content of EPDM to obtain the corresponding PPR/GF/EPDM composites. The compositions of the investigated compounds are summarized in Table 1. The melt blending process was accomplished on an SHJ-25 co-rotating twin-screw extruder (ChengMeng Plastic Machinery Factory, Nanjing, China). The screw speed was 190 rpm. The temperature was 160/170/180/190/190/190/190/190 °C from hopper to die, respectively. The obtained pellets were dried for 8 h at 80 °C in an oven and then processed into a filament with an FLD-25 single-screw extruder (EnBeide Machinery Co., Ltd., Suzhou, China). The screw speed was 600 rpm and the temperature was 180/200/200/210 °C from hopper to die, respectively. The extruded filaments were pulled by a driving wheel and frozen in a water bath. A laser diameter measuring instrument was used to ensure the diameter of filaments was precisely controlled at 1.75  ±  0.1 mm by adjusting the pulling speed. The filaments were dried for another 8 h at 80 °C in an oven before being printed by a HORI Z300 desktop 3D printer (Beijing Huitianwei Technology Co., Ltd., Beijing, China). The printing parameters are listed in Table 2. Samples for tensile strength test, impact strength test, and deformation analysis were printed.

### 2.3. Characterizations

#### 2.3.1. Capillary Rheology Measurements

Pellets processed by the co-rotating twin-screw extruder were used for capillary rheology measurements. The measurements were carried out on a high-pressure capillary rheometer (RH7D, Malvern Instruments Co., Malvern, UK). The temperature was set as 220 °C and shear rate ranged from 100 s^−1^~1000 s^−1^.

#### 2.3.2. Warpage Analysis

**Figure 1:**
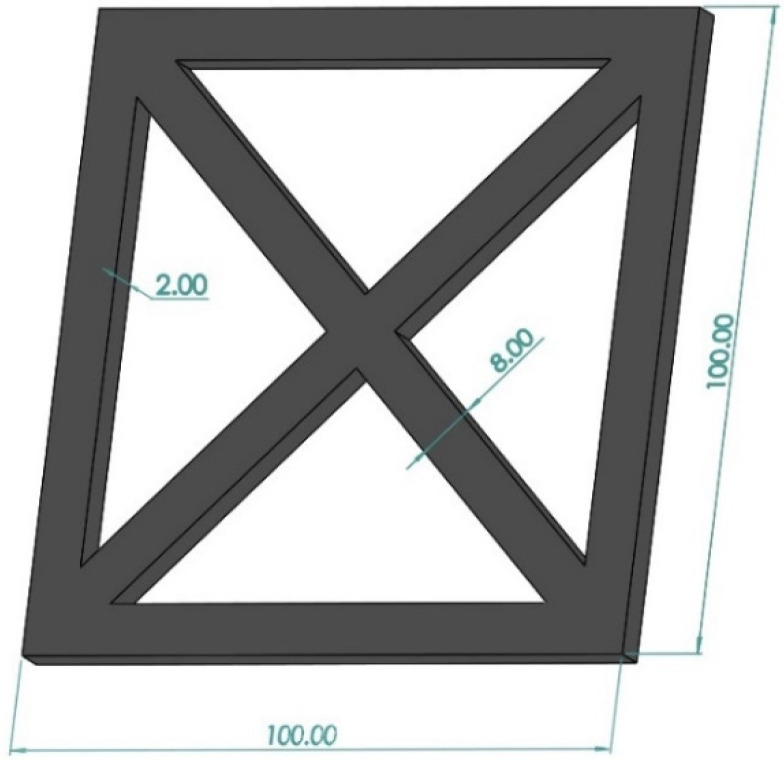
Part designed for warpage analysis. Its dimensions are given in mm.

Figure 1 displays the designed part for characterizing the deformation of different composites. The part was selected as the contraction of the constituting straight sections upon cooling is enhanced by the longitudinal strand orientations and imposes large pulling forces, especially upon the corner areas. Therefore, a more significant deformation at corners is expected to be observed [27]. The printed parts were used for warpage analysis 24 h after fabrication. Warpage analysis was carried out with a 3D scanner (ATOS CORE200 Essential Line, GOM Co., Braunschweig, Germany). The curvature radius of the upper surface of parts was recorded. Warpage curvature was calculated by the equation:

$$k=\frac{1}{r}$$

where k is the warpage curvature, and r is the curvature radius. The value of k reflects the warpage degree of the part. A bigger value of warpage curvature indicates a more serious deformation and vice-versa. The reported value was calculated as the average of five specimens.

#### 2.3.3. Thermal Tests

Thermogravimetric analysis (TGA) was conducted on a thermal gravimetric analyzer (TG209F1, Netzsch Co., Selb, Germany). The heating rate was 10 K/min, and the temperature range was 20–700 °C. Specimens of about 3–6 mg, which were cut from the printed parts, were heated under air flow. The initial decomposition temperature (T_5%_), maximum degradation rate temperature (T_max_), and residual weight were recorded. Derivative weight was calculated as the first derivative of the percentage of residual weight. The initial decomposition temperature and maximum degradation rate temperature were chosen as the temperature when 5% degradation and maximum degradation rate happened, respectively.

DSC measurements were carried out on a differential scanning calorimetry device (Q200, TA Instruments Co., New Castle, DE, USA). Specimens of about 3–5 mg, which were cut from the printed parts, were heated under a dry nitrogen atmosphere. The temperature range was 80–200 °C, and the heating rate was 10 °C/min. All the results were recorded during the first heat process. Sample crystallinity was calculated by the following equation [32]:

$$X_{c}=\frac{\Delta H_{i}}{\Delta H_{i}^{m}\varphi_{i}}$$

where$\;X_{c}$ is the crystallinity of tested sample, $\Delta H_{i}$ is the measured value of fusion enthalpy, and $\Delta H_{i}^{m}$ is the fusion enthalpy of completely crystallized PP and is chosen as 207 J/g in this work [33]. $\varphi_{i}$ means the mass fraction of PP in the tested specimen.

#### 2.3.4. Morphological Observation

Scanning electron microscope (SEM) observation was conducted by an SEM device (Nova NanoSEM 450, FEI Co., Hillsboro, OR, USA). To reveal the morphology of the filaments and the printed parts, the samples were immersed in liquid nitrogen and then brittle fractured. To observe the morphology of EPDM, the corresponding samples were chemically etched in xylene etching solution at 50 °C for 8 h to dissolve EPDM. All specimens were gold-sputtered before SEM observation.

#### 2.3.5. Mechanical Tests

**Figure 2:**
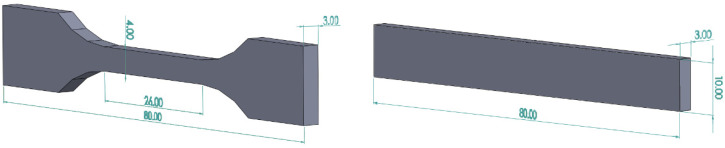
Schematic drawings of the tensile test specimen and notched Izod impact test specimen. The dimensions are given in mm.

Samples for mechanical tests were printed with the FFF printer. Tensile experiments were conducted with an Instron testing machine (Model 5967) at room temperature with a cross head speed of 10 mm/min. The notched Izod impact strength of specimens was measured on a XJUD-5.5 Izod machine (Jinjian Testing Instrument Co., Chengde, China) at room temperature. A 45°V-shape notch (depth of 2 mm) was cut before the test. The shape and dimensions of tensile and impact specimens are displayed in Figure 2. The values of tensile strength, Young’s modulus, elongation at break, and impact strength were calculated as the average of five specimens.

## 3. Results

### 3.1. Rheological Properties

**Figure 3:**
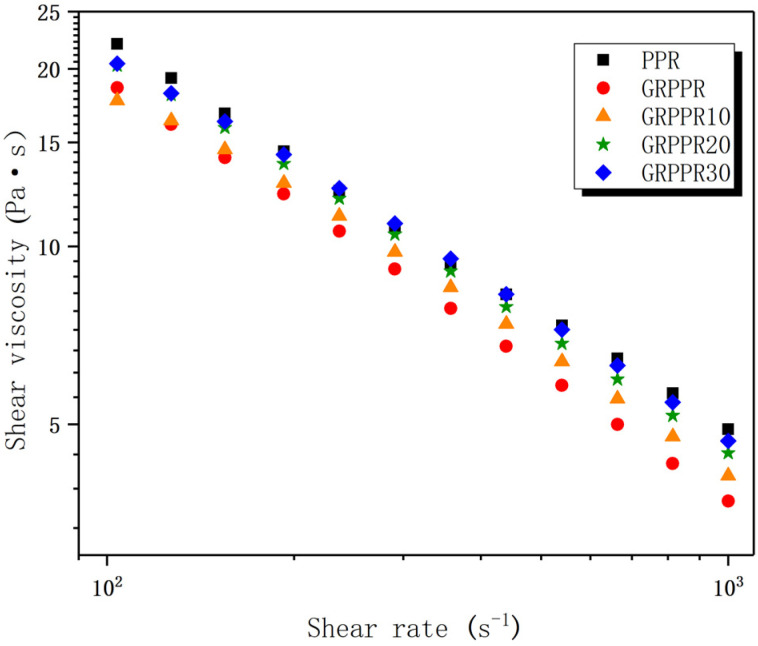
Shear viscosity versus shear rate for various materials measured at 220 °C.

Rheological properties have a significant influence on the processability of material [34]. Favorable melt flow properties are critical for 3D printing quality [35]. Therefore, it is necessary for us to research into the rheology of the material. The shear viscosity at a different shear rate of all the studied materials are shown in Figure 3. One can observe a significant decrease in shear viscosity as shear rate increases from 100 s^−1^ to 1000 s^−1^, which indicates that the materials show a typical shear-thinning behavior. Pure PPR has the highest shear viscosity. When GF are added, the shear viscosity decreases substantially. This is because GF can enlarge the shear effect in its surrounding area, which can help orient the molecular chains [36,37]. The addition of EPDM into the composite causes an increase in shear viscosity, and composites with higher content of EPDM have a more significant increase. This is due to the higher viscosity of EPDM. In addition, GF may collide with the EPDM domains and get deflected in random directions, which can result in increasing the obstruction to shear viscosity of the material [38]. However, even with the highest EPDM content of 30%, the composite still has lower shear viscosity than PPR. Therefore, from the view of rheology, it can be expected that all the composites can be smoothly used for FFF.

### 3.2. Warpage Analysis

**Figure 4:**
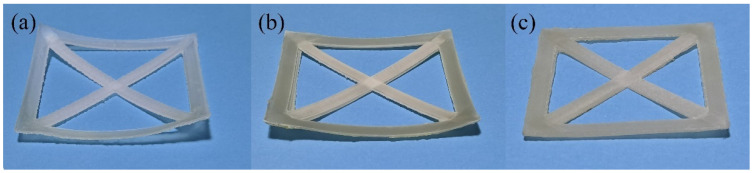
Photos of selected samples for warpage analysis: (**a**) S-PPR; (**b**) S-1; (**c**) S-3.

**Figure 5:**
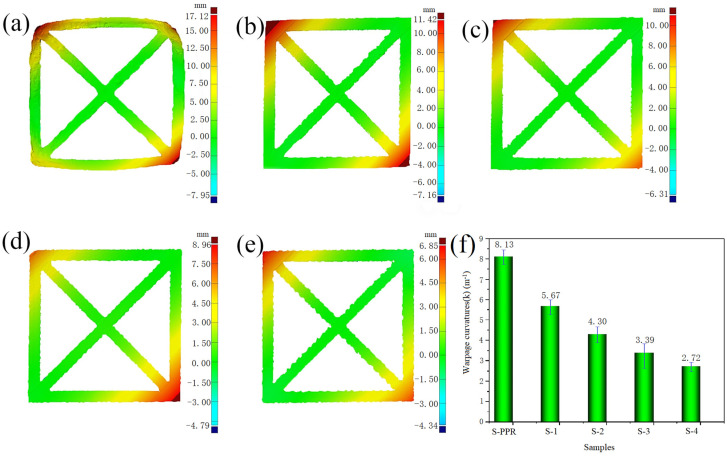
Results of the optical warpage analysis of (**a**) S-PPR; (**b**) S-1; (**c**) S-2; (**d**) S-3; (**e**) S-4; and (**f**) warpage curvatures calculated from (**a**–**e**).

Figure 4 represents the photos of selected printed samples used for warpage analysis, and Figure 5 shows the results of warpage analysis. According to both figures, the reduction of the warpage is significant with the addition of GF and EPDM. S-PPR has a warpage curvature (K) of 8.13 m^−1^, and the warpage curvature of S-1 reduces to 5.67 m^−1^; thus, a 30% reduction is obtained. With the addition of EPDM, the deformation reduces further. The value of K is 4.3 m^−1^ for S-2, and 3.39 m^−1^ for S-3. When the EPDM content reaches 30% (S-4), K is only 2.72 m^−1^. This result shows a 67% reduction compared with S-PPR and a 52% reduction compared with S-1. Meanwhile, from Figure 5a, we can see that the deformation of the part printed with pure PPR is so enormous that a clear square shape can not be observed from a vertical view. With the addition of GF and EPDM, the profile of the parts becomes more and more regular. Finally, a standard square shape can be observed for S-4. Thus, we can conclude that the addition of GF and EPDM can remarkably improve the printability of PPR. The reason is that both GF and EPDM have a lower coefficient of thermal expansion than the PPR matrix. Meanwhile, GF and EPDM as fillers restrict the movement of polymer chains and thus hinder their recovery to a random state, which can lead to higher shrinkage of the material [39,40].

### 3.3. Thermal Behavior

**Figure 6:**
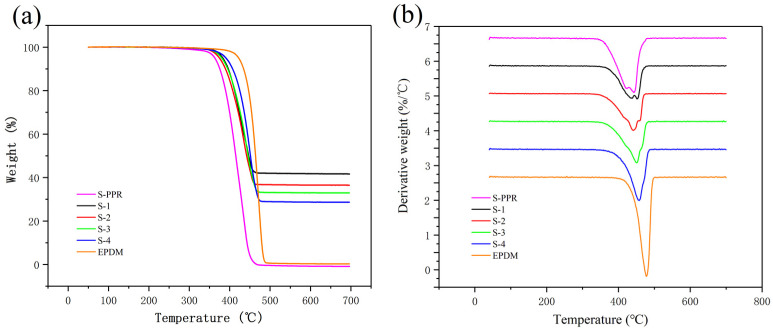
(**a**) Curves of thermal degradation of various materials; (**b**) curves of derivative weight of various materials.

Figure 6 shows the TGA and corresponding derivative thermogravimetric (DTG) analysis curves for different materials in the temperature range of 20–700 °C, and Table 3 lists the corresponding results. It can be found that pure PPR shows the lowest initial degradation temperature (T_5%_) and maximum degradation rate temperature (T_max_), which are 302 °C and 436 °C, respectively. The composites containing GF show a significant improvement in T_5%_ and T_max_. With the addition of EPDM, the temperatures have a relatively complicated change. When the content of EPDM is low, T_5%_ and T_max_ fall slightly from 350 °C and 445 °C of S-1 to 345 °C and 434 °C for S-2, 347 °C and 434 °C for S-3. As for S-4, whose EPDM content is 30%, T_5%_ is 341 °C, which is still lower than S-3. However, its T_max_ reaches 452 °C, which is even higher than S-1. The total weight loss of PPR/GF/EPDM composites rises as the EPDM content increases. The changes are in accordance with the rule of mixture as GF and EPDM have higher thermal stability compared with PPR. Restriction of molecular mobility around GF and EPDM domains is also a reason for the changes [41].

**Figure 7:**
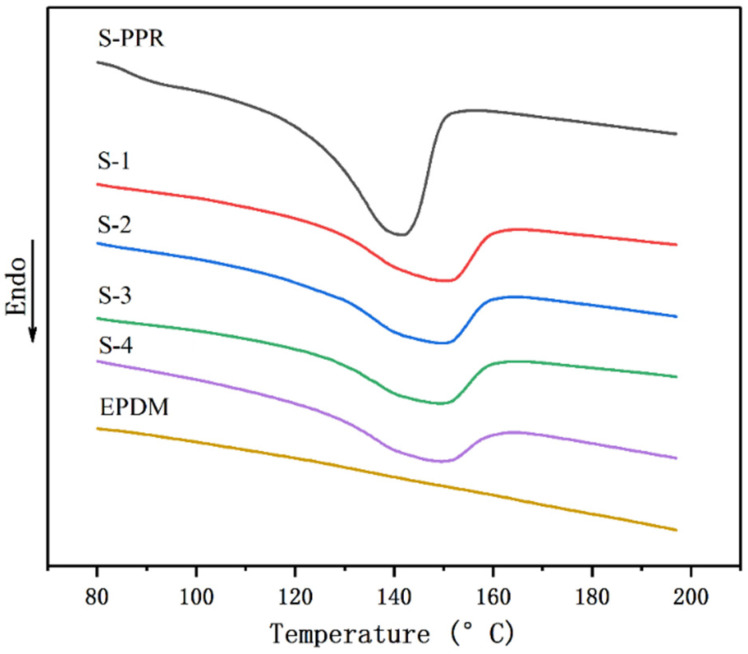
DSC curves of various materials.

Figure 7 shows the DSC curves in the temperature range of 80 °C to 200 °C and Table 4 lists the corresponding results. It can be seen that the melting temperature has a significant increase with the addition of GF, as it rises from 141.1 °C (S-PPR) to 149.4 °C (S-1). The EPDM component causes a slight decrease in melting temperature. The crystallinity of S-PPR is 22.7%, and the GF filler brings a slight increase in crystallinity. With the addition of EPDM, crystallinity further rises, which reaches the highest value of 25.0% when the EPDM content is 10% (S-2). This is because of the heterogeneous nucleation effect of GF and EPDM components [42].

### 3.4. SEM Observation of Filament

**Figure 8:**
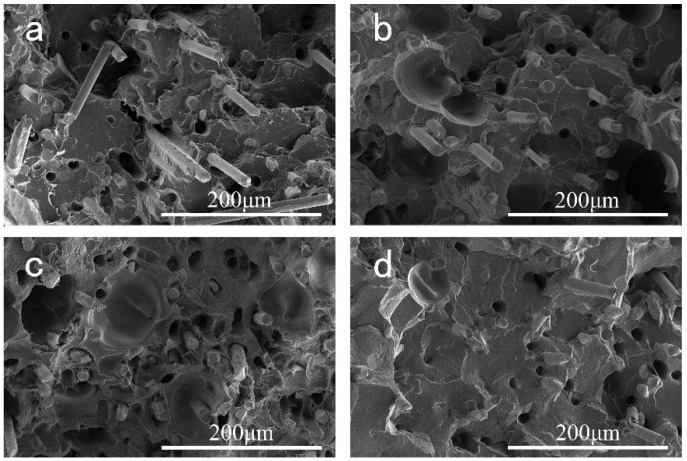
SEM images of cryo-fractured surfaces of different 3D printing filaments: (**a**) S-1; (**b**) S-2; (**c**) S-3; (**d**) S-4. The magnification is 1000.

The SEM micrographs of the brittle fracture surface of the filament are shown in Figure 8. It can be found that the GF are mostly vertical to the surface, which indicates that GF is oriented along the length direction of the filament. A smooth surface of GF can be observed from Figure 8a, and GF remains longer than the other three samples. In addition, we can find a considerable number of holes on the fracture surface, demonstrating the GF that are pulled out from the matrix during the fracture process. This indicates little adhesion between the GF and PPR matrix. In Figure 8b–d, one can observe that, as EPDM content rises, GF length becomes shorter, the interface between GF and PPR matrix becomes vague, and the number of holes decreases, indicating an enhanced interaction between the GF and PPR matrix. This is because the EPDM elastomer may act as the role of sticker and have greater affinity to GF [43]. As the mechanical properties of GF reinforced polymer are mainly determined by the strength and stability of the polymer–fiber interface [44], we can expect the addition of EPDM has a positive effect on the mechanical properties of the material.

### 3.5. SEM Observation of Printed Samples

**Figure 9:**
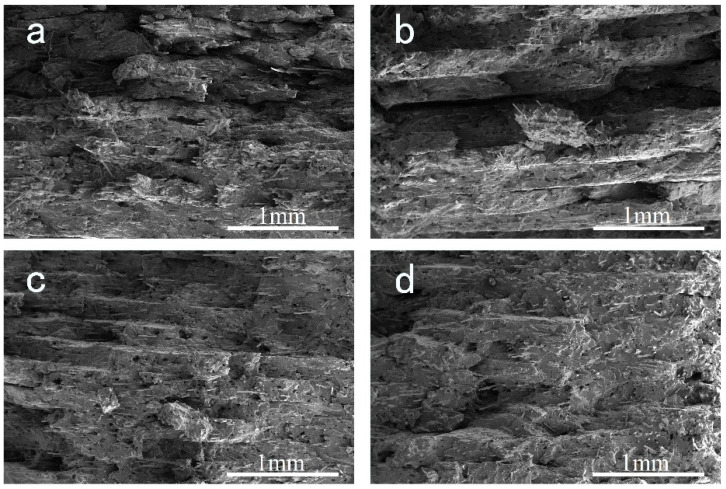
Cross section morphology with low magnification of cryo-fractured samples of (**a**) S-1; (**b**) S-2; (**c**) S-3; (**d**) S-4. The magnification is 130.

**Figure 10:**
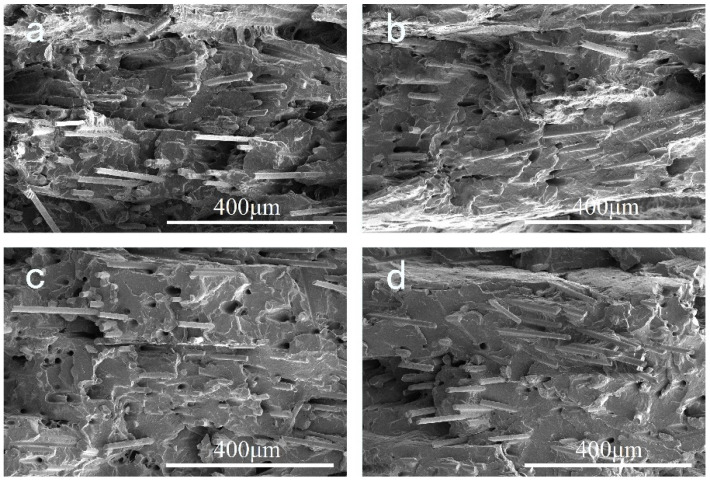
Cross section morphology with high magnification of cryo-fractured samples of (**a**) S-1; (**b**) S-2; (**c**) S-3; (**d**) S-4. The magnification is 500.

Figure 9 shows SEM micrographs of a brittle fracture surface of the FFF printed samples. All the samples present an obvious layered structure. The printed layer thickness is approximately 0.2 mm, which accords with the printing parameter. Adjacent layers are closely stacked and gaps between layers are not observed. No distinct defects can be discovered from the cross section. Figure 10 shows the corresponding SEM micrographs with higher magnification. We can clearly see the alternate ±45° orientation of GF in adjacent layers, which corresponds to the printing manner. It is clear that the adhesion between the GF and PPR matrix enhances as EPDM content increases, which accords with the result of Figure 8 and can be attributed to the same reasons.

**Figure 11:**
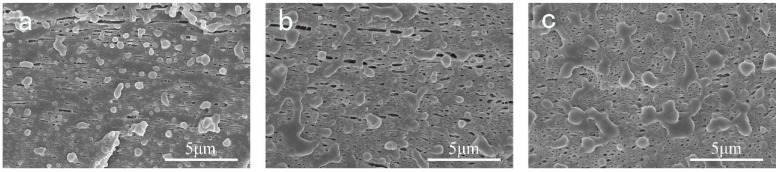
Morphology of dispersed EPDM in the printed samples of (**a**) S-2; (**b**) S-3; (**c**) S-4. The magnification is 21,000.

To study the phase morphology of EPDM, the chemically etched samples were observed. Figure 11 shows corresponding SEM micrographs of brittle fracture surface of the samples. We can observe that EPDM distributes in the matrix evenly. No significant agglomeration can be found. Meanwhile, due to the strong shear and stretching field during the printing process, EPDM exhibits a rod-like shape along the printing direction. In addition, it can be discovered that the aspect ratio decreases as EPDM content increases.

### 3.6. Mechanical Properties

**Figure 12:**
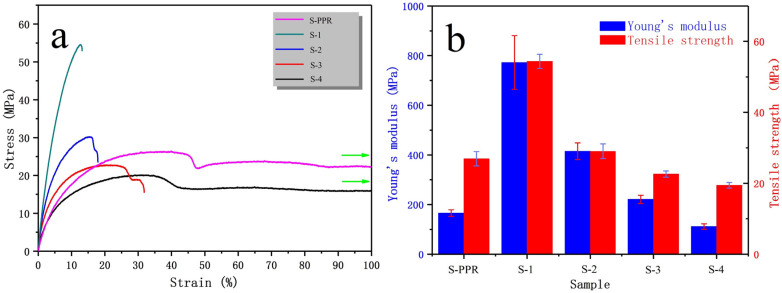
(**a**) Stress–strain curves of different filaments; (**b**) Young’s modulus and tensile strength of different filaments.

The stress–strain curves of filament used for FFF are shown in Figure 12a. Figure 12b shows the corresponding Young’s modulus and tensile strength. From the figure, we can see that GF considerably improves the tensile strength and Young’s modulus of the material. S-PPR filament shows a typical ductile property as the material remains in a yield stage when elongation rate reaches 100%. With the addition of GF, tensile strength increases from 27 MPa to 54 MPa, and Young’s modulus increases from 170 MPa to 770 MPa. However, the elongation at break decreases at the same time. The result shows a ductile-brittle transition [45] happens when GF are added. With the addition of EPDM, the elongation at break improves, indicating that the material becomes ductile again, while the tensile strength and Young’s modulus of the filament decrease. Both EPDM and PP molecules have propyl, thus EPDM can disperse evenly in the PPR matrix, forming sea-island structure. When the composite is impacted by external force, EPDM domains can induce massive crazes and shear zones, absorbing large impact energy [46].

**Figure 13:**
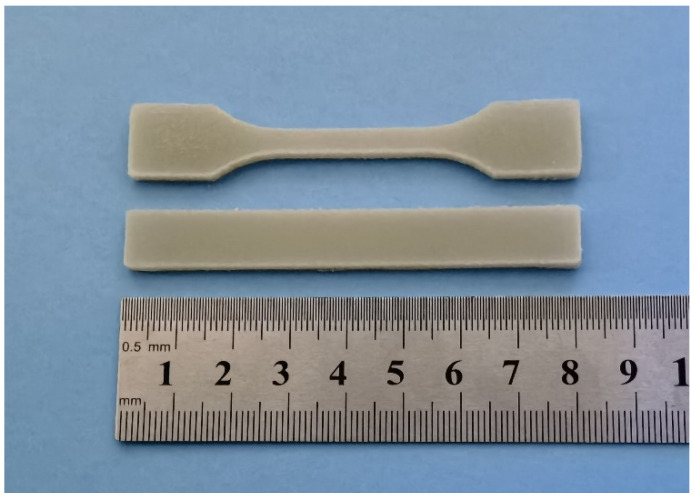
An example of printed specimens (S-3) for tensile and notched Izod tests.

**Figure 14:**
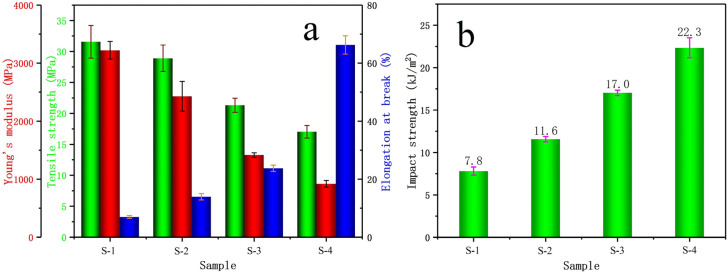
(**a**) Tensile properties of different printed samples; (**b**) Izod impact strength of different printed samples.

Figure 13 presents an example of printed specimens (S-3) for mechanical tests. As can be seen, the specimens have good geometric accuracy. The mechanical properties of the printed samples containing different amounts of EPDM are shown in Figure 14. It should be clarified here that pure PPR can not be printed correctly for mechanical tests due to its serious warpage and shrinkage and therefore there is no result of S-PPR. The results correspond with the results of filament and can be attributed to similar reasons. From the figure, it is clear that a higher content of EPDM leads to a much better toughness. The impact strength of samples rises from 7.8 KJ/m^2^ to 22.3 KJ/m^2^ as EPDM content increases from 0 to 30%, and the elongation at break also rises from 3% to 66%. The increase of impact strength is 186%, and for elongation the increase reaches 2100%. These differences are due to the characteristic of elasticity of EPDM. Meanwhile, the Young’s modulus and tensile strength decrease. However, even for S-4, the tensile strength is still as high as 17 MPa. The decrease is acceptable compared with the large improvement of sample toughness. It should be noted that the mechanical properties of filament and printed samples are different. This is mainly because of the orientation of GF. In the printed samples, GF orientations are ±45°, while GF orients along the length direction in the filament.

## 4. Conclusions

In this work, we use EPDM and GF to modify PPR, expecting to solve the problem of the poor application of PP in FFF. Results show that GF fillers lead to a decrease in shear viscosity of the material while EPDM leads to an increase. However, even with the highest content of EPDM, the shear viscosity is still lower than pure PPR, which indicates that the processed material has good processability. The GF/EPDM modified PPR can largely improve the geometrical accuracy of the printed samples. The warpage curvature decreases from 8.13/m (S-PPR) to 2.72/m (S-4). In addition, compared with pure PPR, the composite has better thermal stability and higher crystallinity. SEM results suggest that the PPR/GF/EPDM composite has better interaction between the GF and PPR matrix. The GF/EPDM modified PPR has significant improvement in toughness, as the elongation at break and impact strength of printed sample increase by 2100% and 186%, respectively. This work has important meaning in expanding the FFF applicable material and in broadening the application of PP.

## Figures and Tables

**Table 1:** Sample designation.

Sample Designation	PPR (wt.%)	GF (wt.%)	EPDM (wt.%)
S-PPR	100	-	-
S-1	60	40	-
S-2	54	36	10
S-3	48	32	20
S-4	42	28	30

**Table 2:** FFF printing parameters.

Printing Parameters	Value
Nozzle temperature (°C)	220
Printing bed temperature (°C)	30
Nozzle diameter (mm)	0.4
Layer thickness (mm)	0.2
Printing orientation (°)	±45
Filling degree (%)	100
Linear printing speed for the 1st layer (mm/s)	72
Linear printing speed for the other layers (mm/s)	80
Contour number	1

**Table 3:** Summary of various degradation temperatures.

Sample	Initial Degradation Temperature (°C)	Maximum Degradation Rate Temperature (°C)
S-PPR	302	436
S-1	351	445
S-2	345	434
S-3	347	444
S-4	341	452
EPDM	374	471

**Table 4:** Summary of thermal properties of various materials via DSC.

Sample	T_m_ (°C)	ΔH_m_ (J/g)	X_c_ (%)
S-PPR	141.4	47.0	22.7
S-1	149.4	28.8	23.2
S-2	148.6	28.0	25.0
S-3	148.2	24.4	24.6
S-4	148.2	24.4	24.6
EPDM	--	--	--

## Data Availability

Not applicable.
